# Efficacy of Ibuprofen Gargle for Postoperative Pain After Mandibular Third Molar Extraction: Protocol for a Phase II, Placebo-Controlled, Double-Blind, Randomized Crossover Trial

**DOI:** 10.2196/35533

**Published:** 2022-05-16

**Authors:** Yasumasa Kakei, Takeshi Ioroi, Takahiro Ito, Yutaro Okazaki, Takumi Hasegawa, Ikuko Yano, Masaya Akashi

**Affiliations:** 1 Department of Oral and Maxillofacial Surgery Kobe University Graduate School of Medicine Kobe Japan; 2 Department of Pharmacy Kobe University Hospital Kobe Japan

**Keywords:** protocol, phase II, placebo-controlled, double-blind, randomized crossover trial, mandibular third molar extraction, pain management, ibuprofen, gargle

## Abstract

**Background:**

Extraction of mandibular third molars is one of the most commonly performed oral surgical procedures, and nonsteroidal anti-inflammatory drugs (NSAIDs) are widely used for pain management. Oral NSAIDs are associated with adverse events such as gastrointestinal disorders, renal and hepatic dysfunction, and platelet dysfunction. Topical analgesics have been proposed as alternatives to oral and injectable medications to safely improve postoperative pain relief. We will conduct a single-center, placebo-controlled, double-blind, randomized crossover trial to assess the pain-relieving effect of an ibuprofen-containing gargle in patients undergoing extraction of mandibular third molars when compared with a placebo gargle.

**Objective:**

This will be the first clinical study to compare the efficacy of an ibuprofen gargle with that of a placebo for relieving postoperative pain in addition to loxoprofen after mandibular third molar extraction.

**Methods:**

This study will be performed at Kobe University Hospital. Participants (N=40) will be randomized equally to 1 of 2 groups. The ibuprofen-placebo group will receive an ibuprofen gargle on postoperative day (POD) 1 and a placebo gargle on POD 2. The placebo-ibuprofen group will receive a placebo gargle on POD 1 and an ibuprofen gargle on POD 2. Both groups will receive ibuprofen gargles on PODs 3-5 at least once daily. The primary objective is to estimate the within-subject difference on a visual analog scale (VAS) before and 5 minutes after using the ibuprofen or placebo gargle on PODs 1 and 2. The secondary objectives are to estimate the within-subject differences in ΔVAS before and 15 minutes after using the ibuprofen or placebo gargle on PODs 1 and 2, ΔVAS before and 5 or 15 minutes after using the ibuprofen gargle on PODs 3-5, overall efficacy (self-completion, 5 scales) on PODs 1-5, daily frequency of use (ibuprofen or placebo gargle and analgesics) on PODs 1-7, and the occurrence of adverse events.

**Results:**

The Certified Review Board of Kobe University approved the study. The intervention was implemented in May 2021. For the primary analysis, we will calculate the mean and SD of ΔVAS_5_ on PODs 1 and 2 and the within-study difference in ΔVAS_5_. The treatment effect will be estimated by dividing the mean ΔVAS_5_ in the within-subject difference by 2 and calculating the *P* value using an unpaired *t* test. For the secondary analysis, we will calculate the mean and SD of ΔVAS_15_ on PODs 1 and 2 and the within-study difference in ΔVAS_15_. The treatment effect will be estimated as in the primary analysis.

**Conclusions:**

This trial will provide exploratory evidence of the efficacy and safety of an ibuprofen gargle for pain reduction after mandibular third molar extraction.

**Trial Registration:**

Japan Registry of Clinical Trials jRCTs051210022; https://tinyurl.com/39ej23zu

**International Registered Report Identifier (IRRID):**

DERR1-10.2196/35533

## Introduction

Extraction of the mandibular third molar is one of the most commonly performed oral surgical procedures [1,2]. Because of the high degree of invasiveness when bone removal and crown division are involved, moderate to severe postoperative complications may occur, such as pain, swelling, difficulty in mouth opening and swallowing [2,3], and postoperative difficulty in oral intake.

Postextraction pain is one of the most common and important postoperative complications after dental extractions, and it is the reason many patients avoid treatment [1]. Nonsteroidal anti-inflammatory drugs (NSAIDs) and acetaminophen are the accepted analgesics for the treatment of postextraction pain [4], and acidic NSAIDs are usually chosen for moderate pain, such as that with mandibular extractions. In patients with gastrointestinal ulcers or aspirin-induced asthma, acetaminophen may be used as an alternative drug [4]. NSAIDs, such as celecoxib [5,6], valdecoxib [7], ibuprofen [8], flurbiprofen [9], lornoxicam [10], etoricoxib [11], and opioid-containing drugs such as oxycodone [12] have been studied to identify the optimal analgesics for pain relief in mandibular third molar extractions. In a systematic review of 21 high-quality clinical trials, Weil et al reported that oral paracetamol (acetaminophen) was safe and effective in the treatment of postoperative pain after extraction of embedded mandibular third molars [13], and in a Cochrane review of 2242 patients, Bailey et al reported that oral ibuprofen was superior to oral paracetamol in the treatment of postoperative pain [14].

Side effects should be considered when prescribing analgesics in the postoperative period, with opioids mainly associated with respiratory depression, nausea and vomiting, and constipation [15], and NSAIDs mainly associated with gastrointestinal disorders, renal and hepatic dysfunction, and platelet dysfunction [16]. To address the problems associated with opioid use, Rindal et al [17] recently published the protocol for a randomized controlled trial of interventions to decrease the prescription of opioids in favor of nonopioid drugs. Additionally, topical use of analgesics has been proposed as an alternative to oral and injectable medications to safely improve postoperative pain relief [9,18,19]. Topical administration has also been reported to reduce side effects without reducing the quality of analgesia [20].

Ibuprofen, developed in the 1960s, is a potent inhibitor of prostaglandin synthesis and reduces fever, pain, and inflammation [21]. Because ibuprofen is pharmacologically active against cyclooxygenase-1 and cyclooxygenase-2, adverse effects such as gastrointestinal and renal dysfunction may occur after systemic administration. However, several reviews and meta-analyses have shown that ibuprofen is effective in adults and children and is the least toxic of the NSAIDs [22-24]. We hypothesized that an oral gargle containing dissolved ibuprofen (0.6% or 1%) delivered directly to the affected area could contribute to pain relief for oral mucositis. In our previous study, we have reported that there are no major safety concerns and that some pain relief is obtained for chemotherapy- or chemoradiotherapy-associated oral mucositis [25].

In postextraction wounds, the loss of keratinized mucosa associated with tooth extraction allows for rapid absorption and efficacy of topically administered medication in the affected area [26]. It is hoped that ibuprofen-containing gargles will provide an efficient drug delivery system targeting the painful area after tooth extraction, with minimal systemic effects. An unresolved limitation of our previous study [25] is that it was not possible to estimate the extent to which a placebo effect may have been present because the study was an uncontrolled trial involving healthy adults and patients with chemotherapy- or chemoradiotherapy-associated oral mucositis [25].

The aim of this clinical study is to evaluate the efficacy and safety of an ibuprofen gargle for relieving postoperative pain in patients with extracted mandibular third molars to assess whether the pain-relieving effect of an ibuprofen gargle can be confirmed without compromising the quality of postextraction pain management in daily practice. To our knowledge, the use of an ibuprofen gargle instead of a placebo for supplemental postoperative pain relief after third molar extractions has not been evaluated previously.

## Methods

### Ethics Approval

The study will be conducted in compliance with the principles of the Declaration of Helsinki (1996), the principles of good clinical practice, and all the applicable regulatory requirements. Ethics approval was provided by the Clinical Research Ethics Committee at Kobe University (reference number: C200024). The trial was registered on the Japan Registry of Clinical Trials (trial registration: jRCTs051210022).

### Consent to Participate

Written informed consent will be obtained from all participants before any study procedure is performed. The participants will have the opportunity to review the consent form and confirm that they fully understand the details of the study procedures. Informed consent will be administered by a suitably qualified and experienced individual who has been assigned this duty by the principal investigator. Secondary use of the data will occur only if the patients provide written informed consent for additional use of their data.

### Primary Objective

The primary objective is to estimate the within-subject difference on a visual analog scale (VAS) that ranges from 0 (no pain) to 10 (worst pain) continuously immediately before and 5 minutes after the use of an ibuprofen or placebo gargle on postoperative days (PODs) 1 and 2 in patients undergoing mandibular third molar extractions (ΔVAS_5__ibuprofen − ΔVAS_5__placebo).

### Secondary Objectives

The secondary objectives are to evaluate the within-subject difference in ΔVAS 15 minutes before and 15 minutes after use of an ibuprofen or placebo gargle (ΔVAS_15__ibuprofen − ΔVAS_15__placebo) on PODs 1 and 2, the ΔVAS before and after 5 or 15 minutes of the use of an ibuprofen gargle on PODs 3-5, the overall efficacy (self-completion, 5 scales) on PODs 1-5, the daily frequency of use (ibuprofen or placebo gargle and analgesics) on PODs 1-7, and the occurrence of adverse events.

### Study Design, Setting, and Population

**Figure 1:**
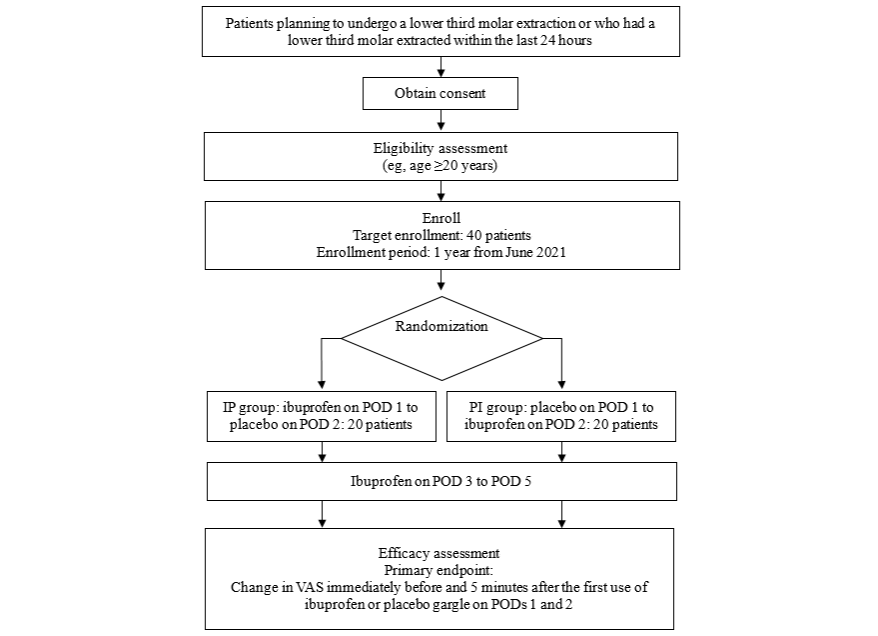
Flowchart of the study design. IP: Ibuprofen-Placebo; PI: Placebo-Ibuprofen; POD: postoperative day; VAS: visual analog scale.

This study is designed as a single-center, placebo-controlled, double-blind, randomized crossover trial. The patient flowchart is shown in Figure 1. This study will be performed at Kobe University Hospital. All study data will be stored and archived in the data center at Kobe University Hospital. We will use Research Electronic Data Capture (REDCap), which is an electronic data system for clinical research, to manage the data and protect confidentiality before, during, and after the trial. The study population will comprise patients who undergo third molar extraction and will be selected based on the inclusion and exclusion criteria given in Textbox 1. All patients undergoing third molar extraction during the study period (June 1, 2021, to September 30, 2022 [expected end date]) will be provided with information about the study. All patients will be asked if they wish to participate in the study and will be enrolled after obtaining written informed consent.

**Inclusion and exclusion criteria.**

**Inclusion criteria**

- Patients planning to have a mandibular third molar extraction or who have had a mandibular third molar extracted within the last 24 hours
- Patients aged 20 years or older at the time of consent acquisition
- Patients for whom documented consent has been obtained regarding their voluntary participation in this clinical study

**Exclusion criteria**

- Patients with peptic ulcers
- Patients with concurrent severe or uncontrolled concomitant medical conditions
- Patients with a history of hypersensitivity to any component of the ibuprofen gargle
- Patients with impaired cardiac function or clinically significant heart disease
- Patients with aspirin-induced asthma
- Patients who use analgesic drugs at least once a week for any chronic pain
- Patients with dementia, psychiatric symptoms, drug addiction, or alcoholism
- Pregnant or lactating women
- Patients deemed inappropriate (for miscellaneous reasons) based on the assessment of the investigator or the subinvestigator

### Surgical Procedure and Prohibited Interventions

All surgeries should be performed by an experienced oral surgeon with a minimum of 3 years of postgraduate experience using the same protocol. Povidone iodine solution will be applied inside the mouth, and 2% lidocaine + 1:80,000 epinephrine carpules will be used to block the inferior alveolar nerves by injection. A mucoperiosteal envelope flap will be created using a standard incision. If needed, bone removal and tooth sectioning will be performed with a low-speed hand piece under sufficient sterile solution irrigation. Following tooth removal, the socket will be irrigated with 10-20 mL of saline, and the flap will be sutured using 2-3 resorbable sutures (3–0 Vicryl; Ethicon Inc). The operation time will be recorded in minutes from the time of incision to the completion of the last suture. If needed, supplementary intraoperative local analgesia will be administered and recorded. Loxoprofen (60 mg) will be prescribed postoperatively, and the patients will be instructed to take up to 3 tablets daily for pain. The dosing information will be recorded in the patient diary with the number of analgesics used per day for up to 10 days postoperation. Antibiotics will be prescribed postoperatively and will be administered up to POD 2. Patients will be prescribed 1 loxoprofen sodium tablet (60 mg) daily and the ibuprofen and placebo gargles as part of the study protocol. The use of other analgesics, such as opioids and other NSAIDs, is prohibited.

### Ibuprofen and Placebo Gargle Intervention and Treatment Protocol

The ibuprofen gargle is manufactured at the Department of Pharmacy, Kobe University Hospital. The gargle (100 mL) contains ibuprofen 600 mg (0.6%), sodium hydroxide, sodium hydrogen carbonate, hydrochloric acid (to regulate pH), glycerin, and flavor. The placebo gargle formulation is the same but without ibuprofen. The intervention or treatment protocol will be as follows: (1) ibuprofen-placebo (IP) group prescribed with ibuprofen gargle on POD 1, placebo gargle on POD 2, and ibuprofen gargle on PODs 3-5 at least once daily and (2) placebo-ibuprofen (PI) group prescribe with placebo gargle on POD 1, ibuprofen gargle on POD 2, and ibuprofen gargle on PODs 3-5 at least once daily.

As for the dosage, approximately 10 mL of gargle solution is dispensed into a measuring cup and retained in the mouth in contact with the affected area for at least 30 seconds (preferably 1 minute), and it is then discarded.

Patients will not drink water or rinse their mouths for at least 5 minutes after using the gargle.

As a general rule, patients will be allowed an interval of at least 15 minutes between uses of the study drug. The maximum daily dosage should be one bottle (100 mL).

### Randomization (Allocation)

Participants will be randomly assigned to either the IP group or the PI group with a 1:1 allocation using the permutation random block method; they will be stratified by category according to whether a maxillary third molar is to be extracted in addition to a mandibular third molar. The block size will not be disclosed to ensure that blinding is maintained. The allocation sequence for the randomization method will be generated by the biostatistician. The trial participants, care providers, and outcome assessors will be blinded. Because this is a crossover study in which both groups receive the actual drug, an unblinding procedure will not be incorporated into the study.

All subjects providing consent to participate, fulfilling the inclusion criteria, and not meeting any of the exclusion criteria will be randomized. The principal investigator or subinvestigator will send a subject enrollment form by email to the data center. The staff at the data center will confirm the eligibility of the participants and issue them the enrollment confirmation form that contains the eligibility judgment, randomization assignment result from the generated random sequence, and enrollment number. The form will then be sent to the principal investigator or subinvestigator.

### Primary End Point and Secondary Efficacy and Safety End Points

The primary end point is the change in the within-subject VAS immediately before and 5 minutes after the use of the first ibuprofen or placebo gargle on PODs 1 and 2 (ΔVAS_5__ibuprofen − ΔVAS_5__placebo and ΔVAS_5__placebo − ΔVAS_5__ibuprofen).

The secondary efficacy end points are the following:

1. Δwithin-subject VAS before and 15 minutes after the first use of the ibuprofen or placebo gargle on PODs 1 and 2 (ΔVAS_15__ibuprofen – ΔVAS_15__placebo)

2. ΔVAS before, and 5 and 15 minutes after the use of the first ibuprofen gargle on PODs 3 through 5

3. Overall daily efficacy on PODs 1 through 5

4. Number of uses of the gargle and analgesic drug per day on PODs 1 through 7

As a strategy to improve adherence to the intervention protocols, the ibuprofen and placebo gargles will be prepared according to each POD (5 bottles, 1 for each POD from 1 to 5). The patients will return the bottles of gargle at the end of the treatment period; the amount of any remaining solution will be measured and recorded.

The secondary end point for safety is the presence or absence of adverse events associated with the conduct of this clinical study.

### Time Schedule of Intervention, Outcomes, and Other Assessments

The relationships between the interventions, outcomes, other assessments, and visits associated with the participants of this study are shown in Table 1.

**Table 1:** Summary of study assessments and procedures.

Time point		Study period								
		Enrollment	Allocation	Postallocation						Close-out
		POD^a^ 0 (pre-extraction)	POD 0 (postextraction)	POD 1	POD 2	POD 3	POD 4	POD 5	PODs 6 to 10	
**Enrollment**										
	Eligibility screen	✓								
	Informed consent	✓								
	Registration	✓								
	Surgical information^b^	✓	(✓)^c^							
	Allocation		✓							
**Interventions**										
	I^d^ then P^e^			✓						
	P then I				✓					
	I					✓	✓	✓		
**Assessments**										
	VAS^f^ (5 min)			✓	✓	(✓)	(✓)	(✓)		
	VAS (15 min)			✓	✓	(✓)	(✓)	(✓)		
	Diary^g^			✓	✓	✓	✓	✓	✓	
	Adverse events			✓	✓	✓	✓	✓	✓	

^a^POD: postoperative day.

^b^Sex, age, reason for extraction, side of extraction, Pell-Gregory classification, Winter's classification.

^c^items collected on a voluntary basis and not mandatorily.

^d^I: ibuprofen.

^e^P: placebo.

^f^VAS: visual analog scale.

^g^Total amount of rescue medications, number of tablets, number of analgesics used (gargles), self-reported global efficacy.

### Participant Retention

Following tooth extraction and suturing, an appointment will be scheduled for suture removal approximately 1 week after the procedure. We believe that scheduling the suture removal appointment immediately postoperatively will help patients comply with the protocol. Data from participants who discontinue their participation in the study or those who deviate from the protocol will be included in the full analysis set.

### Data Collection and Management

The primary investigator or subinvestigator will enter the case report form (CRF) data for each participant into the electronic data capture system. The principal investigator will confirm that the entered CRF data are complete and correct, electronically sign the CRF in the electronic data capture system, and then obtain a printout of the signed CRF for filing. The CRF printout will be retained. If there are any queries about the CRF data that are entered by the staff at the data center, the primary investigator or subinvestigator should respond promptly to these queries. Only the biostatistician will have access to the final data set.

### Statistics

#### Sample Size Calculation

The target number of subjects is 40, with 20 subjects in the IP group and 20 in the PI group. In a previous report involving healthy subjects and patients with chemotherapy- or chemoradiotherapy-associated oral mucositis [25], the mean ΔVAS and SD of pain relief after 3 days of use were −1.28 and 0.84 (n=7 patients), respectively, of which the preuse VAS value for the ibuprofen gargle was >3. In the subgroup with a VAS≥3 before ibuprofen gargle use, the ΔVAS was −1.56, and the ΔSD was 0.81 (n=5).

In this study, we estimate a ΔVAS_5_ of −1.5 and ΔSD of 1.2 for ibuprofen gargles, and a ΔVAS_5_ of −0.7 and ΔSD of 1.2 for placebo gargles, assuming a placebo effect of less than half the ΔVAS_5_ of ibuprofen gargles. Therefore, for obtaining a mean within-subject difference of 0.8 in ΔVAS_5_ (ΔVAS_5__ibuprofen − ΔVAS_5__placebo), a common ΔSD of 1.2, a ratio of 0.8-1.2 for between- and within-subject variance, an alpha error of .05, and a beta error of .1, a total of 30 cases are required. Hence, considering a potential withdrawal rate of 25%, we plan to enroll 40 patients (20 per group).

No additional analyses (eg, subgroup and adjusted analyses) will be performed.

#### Analysis

A summary of the planned statistical analysis for this study is provided below. The final analysis will be performed after data from the subjects are obtained and locked at the end of the follow-up period.

The full analysis set is the set of randomized subjects who receive at least 1 dose of the study drug and excludes those without baseline data or with major protocol violations (eg, absence of informed consent or enrollment outside the study period). The per protocol set is the subset of subjects in the full analysis set who comply sufficiently with the protocol and excludes those associated with any of the following: violation of the inclusion criteria, violation of the exclusion criteria, and missing primary end point data.

The safety analysis set is the set of subjects who receive at least 1 dose of the study drug.

#### Handling of the Data

If there is any doubt about the data summarization or analysis, the biostatistician and the study representative will discuss the issue and decide how it will be addressed. When missing data are identified, the researcher will contact the subject. If the within-subject difference or ΔVAS cannot be calculated owing to missing VAS values, the within-subject difference or ΔVAS at that time will be recorded as 0. If any missing or deficient values other than the VAS values are not obtained, no analysis will be performed.

#### Primary Analysis

In this study, the mean and SD of ΔVAS_5_ on PODs 1 and 2 and, where appropriate, the 95% CI of the mean will be calculated. We will also calculate the mean and SD of the within-study difference in ΔVAS_5_ and, where appropriate, the 95% CI of the mean. The treatment effect will be estimated by dividing the mean of ΔVAS_5_ in the within-subject difference by 2 and calculating the *P* value using an unpaired *t* test.

#### Secondary Analysis

The mean and SD of ΔVAS_15_ on PODs 1 and 2 and, where appropriate, the 95% CI of the mean will be calculated. We will also calculate the mean and SD of the within-study difference in ΔVAS_15_ and, where appropriate, the 95% CI of the mean. The treatment effect will be estimated by dividing the mean of ΔVAS_15_ in the within-subject difference by 2 and calculating the *P* value using an unpaired *t* test.

The mean and 95% CIs of ΔVAS_5_ and ΔVAS_15_ for PODs 3-5 will be calculated for each group. Analysis of covariance with the preuse VAS value or the strata factor as a covariate will be performed to calculate the adjusted ΔVAS_5_ and ΔVAS_15_ and their 95% CIs for each group. We will calculate summary statistics for the overall daily efficacy on PODs 1-5, scale by scale and group by group. Summary statistics will be calculated for each group for the number of times ibuprofen or placebo-containing products and analgesics are used on PODs 1-7.

### Adverse Events

We define an adverse event as any disease, disability, death, or infection that occurs during this study. Adverse event monitoring will begin on POD 1 and continue up to POD 5, inclusive. The principal investigator or subinvestigator will record all the adverse events in the CRF and treat and monitor the patient until resolution during the study. If the principal investigator or subinvestigator finds a potentially causal relationship between the adverse event and the study drug, all adverse events will be recorded for reporting to the review board. This study is insured for clinical trials, with up to approximately US $385,000 guaranteed for death cases.

### Monitoring for Compliance With Human Rights and Welfare

Periodic monitoring of the study will be performed to confirm if the human rights and welfare of the subjects are being protected, the study is being conducted safely in accordance with the protocol and the applicable regulatory requirements under the Clinical Trials Act, and the data are being collected properly. The principal investigator will appoint a responsible monitor and other monitors for the study. The items to be checked during monitoring are specified in the written procedure for monitoring the study. Any changes required by the ethics committee will be communicated to the participants by the investigators.

For quality assurance, the study will be examined to determine if it is being conducted in accordance with the protocol and written procedures, independently and separately from the routine monitoring activities.

## Results

Participant recruitment began on June 1, 2021. The expected date of completion (last visit of the last patient) is September 30, 2022. All data acquired during the study period will be analyzed. Additionally, as this is a short-term trial performed over 7 days, an evaluation of the interim results is not planned.

This manuscript is based on the current version of the study protocol (version 1.1, last updated on March 1, 2021). The study was first authorized on March 1, 2021. The results of this study will be available on the Japan Registry of Clinical Trials website. The results will also be disseminated via presentations at regional and international conferences, such as those for dentistry and oral and maxillofacial surgery. The results will also be submitted for publication in a peer-reviewed journal.

## Discussion

### Significance of the Study

This single-center, placebo-controlled, double-blind, randomized crossover trial will be the first well-designed clinical study to evaluate the efficacy of an ibuprofen gargle for relieving postoperative pain after extraction of mandibular third molars. Postoperative pain management after third molar extraction is essential, and NSAIDs are the first-line treatment. To address the side effects of oral NSAIDs, this study will compare a topical ibuprofen gargle with a placebo. Previous studies have not compared the use of ibuprofen and a placebo. The anticipated findings in this study are that the topical ibuprofen gargle will be more effective than the placebo in controlling postoperative pain after third molar extraction.

### Overview

Mandibular third molars are commonly extracted, and postoperative pain is a major complication resulting from the extraction. Symptomatic pharmacological treatment aims to provide postoperative pain relief. Continuous medication with NSAIDs is recommended as the first-line treatment after extraction of mandibular third molars to relieve pain and inflammation. Oral administration of NSAIDs can lead to various adverse effects; therefore, topical NSAIDs are preferred to minimize these side effects [9,27,28]. The problem with the administration of NSAIDs thrice a day is that the analgesic effect wears off and postextraction pain recurs. Several methods of treating intermittent pain with analgesic rinses have been reported in the past, but none have been approved in Japan [18,27,28]. Additionally, to date, there have been few well-designed clinical studies evaluating the efficacy and safety of analgesic gargles to relieve postoperative pain after mandibular third molar extraction. Thus, recent studies have focused on the development of topically administered NSAIDs in the form of gels, toothpastes, and rinses [27,28]. However, few studies have highlighted the benefit of ibuprofen gargles for postoperative pain after tooth extraction. We believe that an ibuprofen gargle can be used after third molar tooth extraction to provide seamless pain relief between doses of NSAIDs when additional pain relief is required before the next dose of NSAIDs is due.

### Safety Concerns

Regarding the safety concerns associated with the ibuprofen gargle, the gargle is retained in the mouth for only approximately 1 minute before being discarded. Therefore, systemic absorption of ibuprofen is unlikely. Moreover, the concentration to be used in this study (600 mg ibuprofen/100 mL) is equal to the maximum daily allowance (600 mg) approved for oral ibuprofen, and if used according to the recommended method, the amount of drug that could be ingested accidentally is less than the maximum daily allowance. Therefore, adverse events due to orally absorbed ibuprofen are less severe than or identical to those reported for oral ibuprofen.

### Rationale

For ethical reasons, only ibuprofen-containing gargles will be used from POD 3. The reason for the crossover study between POD 1 and POD 2 was that the median duration of the effect of the ibuprofen-containing gargle was approximately 20 minutes in our previous study, and there was no carryover effect when comparing POD 1 and POD 2 [25]. Therefore, we chose an ibuprofen gargle as the test drug for treating patients after mandibular third molar extraction and decided that a period of 5 days was sufficient for a short-term treatment study. Adverse events and all conditions that occur will be recorded and observed until the conditions subside within the study period, regardless of whether there is a causal relationship with the clinical study.

### Strengths

Because previous clinical trials used only ibuprofen-containing drugs without a placebo or control group, this clinical trial is designed as a placebo-controlled comparative study to examine the placebo effect. Therefore, this study will compare an ibuprofen gargle with a placebo for treating postoperative pain after mandibular third molar extraction; it will be performed as a phase II, placebo-controlled, double-blind, randomized crossover trial.

### Limitations

The main limitation of this study is the short duration (7 days).

### Conclusions

Postoperative pain management after third molar extraction is essential, and NSAIDs are the first-line treatment. To address the side effects of oral NSAIDs, this study will compare a topical ibuprofen gargle with a placebo. The results of this study could provide valuable evidence to support the use of an ibuprofen gargle for patients after mandibular third molar extraction. In addition to NSAIDs, ibuprofen gargles may relieve the pain caused by mandibular third molar extraction. This study fills a gap in the literature and may lead to a safer, but still effective, pain relief option to improve patients’ postoperative comfort.

## Abbreviations

CRF: case report form
IP: ibuprofen-placebo
jRCT: Japan Registry of Clinical Trials
NSAID: nonsteroidal anti-inflammatory drug
PI: placebo-ibuprofen
POD: postoperative day
VAS: visual analog scale
